# Allergic Signs in Glioma Pathology: Current Knowledge and Future Perspectives

**DOI:** 10.3390/cancers11030404

**Published:** 2019-03-22

**Authors:** Massimo Costanza, Gaetano Finocchiaro

**Affiliations:** Unit of Molecular Neuro-Oncology, Fondazione IRCCS Istituto Neurologico Carlo Besta, 20133 Milan, Italy; gaetano.finocchiaro@istituto-besta.it

**Keywords:** allergy, glioma, GBM, IgE, IL-4, mast cells

## Abstract

Historically restrained to immune defense against parasite infections, allergic inflammation has been recently rediscovered to protect from a wide array of environmental triggers, such as xenobiotics and carcinogens, which can induce DNA damage and ultimately lead to cancer development. Moreover, cells and mediators typical of allergic responses can importantly modulate the tissue inflammatory milieu, which represents a crucial gatekeeper towards the acquisition of malignancy by cancer cells through immune escape. Numerous studies have described an inverse association between allergies and glioma development. Mast cells, key players of allergic reactions, have been recently found at increased numbers in glioblastoma multiforme (GBM), the most common and lethal primary brain tumor, and they have been implicated in GBM pathogenesis. In this review, we summarize epidemiological studies and discuss the main evidence highlighting a potential interplay between allergic responses, and glioma formation and progression. Last, we draw future lines of research for better clarification whether and through which mechanisms allergic inflammation might impact on gliomagenesis. The comprehension of the immune mechanisms favoring or counteracting tumor growth might open the path to novel immunotherapy approaches.

## 1. Introduction

Gliomas are the most common primary brain tumors of adults and are classified into different types and grades, based on histologic and molecular patterns [1]. Glioblastoma multiforme (GBM) accounts for approximately 70% of malignant gliomas and it is characterized by severe morbidity and high mortality [2]. Recent data suggest that GBMs arise from driver mutations of neural stem cells in the subventricular zone of the adult human brain [3]. Divergent genetic evolution may then be one factor underlying GBM progression [4]. The best-established risk factor for malignant gliomas is the exposure to ionizing radiation, however, a large body of literature has suggested that subjects with atopy have a diminished probability of developing gliomas [2]. The reason for this inverse association is still unknown. Type 2 immune reactions have long been ascribed to host defense against parasite infections, with allergic inflammation considered as an off-targeted side-effect of these protective responses [5]. Interestingly, recent lines of evidence have clearly shown that type 2 immunity exerts crucial functions in a complex array of responses, including venom detoxification as well as protection from hematophagous fluids, toxic xenobiotics and carcinogens [5]. These responses are driven by T helper (Th)2 cells, which produce mainly interleukin (IL)-4 and IL-13, that in turn promote immunoglobulin (Ig)E production by B cells, and IL-5, important mediator for eosinophil activation [6]. Allergic inflammation includes also IgG1 and components of innate immunity, such as mast cells, basophils, eosinophils, alternatively-activated macrophages and innate lymphoid cells [5].

The relationship between allergic inflammation and cancer has been the subject of several investigations, leading to opposite views regarding the impact of these responses on tumor growth. According to one current of thought, allergic reactions might foster tumor development by impairing Th1 and cytotoxic T cell functions, and by supporting myeloid cells with a suppressive phenotype [7]. Indeed, in a model of breast cancer, IL-4-producing Th2 cells have been shown to sustain metastasis by promoting effector functions of tumor-associated macrophages [8]. Conversely, according to an alternative viewpoint, allergic responses might be important for tumor-surveillance [5]. In a model of skin cancer induced by exposure to the carcinogen dimethylbenz[a]anthracene (DMBA), it was demonstrated that IL-4-deficient mice develop tumors of increased size and greater incidence compared to wild-type mice [9]. Moreover, sensitization to DMBA was associated with the establishment of a strong autoreactive IgE response that was protective against carcinogenesis [9]. A similarly puzzling scenario emerges from studies analyzing the contribution of mast cells to cancer, as they have been suggested as either protective or detrimental [10]. Most probably, depending on the specific neoplastic condition and the experimental model utilized, allergic inflammation can promote or counteract tumor development [10]. Very recently, the long controversy regarding the activity of eosinophils in tumor development has gained new insight from the study of Holland and colleagues [11,12]. In this work, it has been shown that in several solid tumors such as hepatocellular carcinoma, breast cancer and prostatic carcinoma, eosinophils can dampen tumor growth by inducing tumor cell-cytotoxicity. In particular, tumor-derived IL-33 was shown to promote chemokine CCL11-mediated eosinophil infiltration and degranulation [11,12].

In recent years, numerous studies have investigated the contribution of allergic components in the pathogenesis of gliomas and GBM. In this review, we summarize main epidemiological, histopathological and genetic studies that have suggested the involvement of allergic inflammation in glioma pathology and we discuss experimental approaches to further characterize in future the relationship between this specific arm of the immune system and brain tumorigenesis.

## 2. Epidemiology of Gliomas and GBM in Allergic Subjects

Most of the research on glioma and allergy has relied on epidemiological investigations, which have shown an inverse association between allergies and the risk of developing gliomas since the early 1990s [13]. In an international population-based case-control study performed in six different countries and including 1178 cases, it was reported that there is a reduced risk of 30–40% of developing gliomas among subjects with asthma, eczema and other allergic conditions [14]. In two subsequent case-control studies performed by Wiemels and colleagues on subjects enrolled in the San Francisco Bay Area, a significant inverse association for both self- and proxy-reported histories of allergic conditions with a diagnosis of adult glioma was shown [15,16]. A particularly decreased risk was found among subjects with allergies to pollen, dairy products and nuts [15]. Additionally, a strong association with diminished risk of developing gliomas has been detected in cases with late-onset (>12 years) respiratory allergies [16]. Another case-control study enrolling 489 cases of gliomas among three different hospitals in the United States (US), confirmed an inverse association between the risk of glioma and history of any allergy (e.g., eczema, allergy to insects, chemicals, etc.) [17]. Of note, in this work, people affected by both allergies and autoimmune diseases were at particularly low risk of developing glioma (odds ratio (OR) = 0.24, 95% CI, 0.14–0.42) [17]. In addition, a retrospective study has shown in two independent cohorts selected from Swedish twin registries that high-grade gliomas (grades III and IV) are inversely associated with allergic conditions that comprised of eczema, asthma, hay fever, and allergic rhinitis (hazard ratio, HR = 0.45 and 0.46, 95% CI) [18]. In a large, population-based case-control study in the UK, history of asthma, eczema, hay fever or other types of allergy was inversely correlated to the risk of developing glioma (OR = 0.63, 95% CI, 0.53–0.76) [19]. Association with allergies was not increased or decreased in grade I/II compared with grade III/IV tumors in this work [19]. Moreover, there was no evidence for a gradient of risk with the age of onset, time since onset or with the number of conditions presented [19]. A meta-analysis of both case-control and cohort studies published between 1979 and 2007 and including 3450 cases, has shown that the risk of glioma was decreased by 40% among subjects with a history of allergy, 30% diminished among those with a history of eczema, and 30% reduced among those with a history of asthma [20]. Similar findings have been obtained in a large population-based case-control study published in 2007 and comprising 1527 cases of gliomas recruited in Denmark, Norway, Finland, Sweden and Southeast England [21]. This epidemiological trend has been confirmed also in another case-control study involving 366 cases of glioma recruited among the German population [22]. In an alternative approach, Il’yasova and colleagues have compared 388 glioma cases with three different groups of control—siblings, friends and clinic-based controls—to better evaluate the contribution of genotypic (for siblings) or environmental (for friends) effects on the relationship with allergic inflammation [23]. Allergies were always found significantly inversely associated with gliomas: ORs were 0.53 (95% CI, 0.15–1.84), 0.54 (95% CI, 0.28–1.07) and 0.34 (95% CI, 0.23–0.50) with siblings, friends and clinic-based controls, respectively [23]. A case-control study recruited 855 high-grade glioma patients from four different geographic regions of the US and belonging to five inherited glioma risk variants: 5p15.3 (*TERT*), 8q24.21 (*CCDC26/MLZE*), 9p21.3 (*CDKN2B*), 11q23.3 (*PHLDB1/DDX6*) and 20q13.3 (*RTEL1*) [24]. A significantly stronger inverse association of allergy history with glioma was detected among subjects who do not carry the glioma risk allele in the 9p21.3 region, while it was suggestively higher among those carrying the glioma risk allele in the 20q13.3 region [24]. In 2014, Cahoon and co-workers analyzed a cohort of 4.5 million male US veterans comprising 4383 cases of patients with a discharge diagnosis of malignant neoplasm of the brain, which included mainly gliomas, expected to cover 95% of cases, and some rare childhood tumors such as medulloblastoma. In this study, allergy/atopy of long latency (≥10 years) was associated with a reduced risk of developing brain cancer (rate ratio = 0.60, 95% CI, 0.43–0.83) [25]. Last, a recent multicenter case-control study carried out in four areas in France in 2004–2010 has explored the relationship between allergy (e.g., asthma, eczema, rhinitis/hay fever and other allergic conditions) and the risk of glioma in 273 glioma cases and 982 matched controls [26]. In addition to confirming previous findings, this work highlighted a dose-effect relationship between the number of allergic conditions and the inverse association with glioma risk and a stronger relationship in women [26]. To avoid the possibility that allergic inflammation might be somehow influenced by (or be the effect of) brain tumor itself, Schwartzbaum and colleagues have investigated germline polymorphisms as potential biomarkers of GBM susceptibility, selecting five single nucleotide polymorphisms (SNPs) strongly associated with asthma and allergic conditions [27]. Authors found that in subjects carrying three of these polymorphisms, located on the *IL-4 receptor α* (*IL4RA*) and *IL13* genes, the odds ratios for GBM were in the opposite direction with those for asthma [27]. Interestingly, pre-diagnostic serum levels of IL-4 and soluble IL-4RA have been later found inversely associated with gliomas and GBM in a nested case-control study including 487 glioma cases and 487 matched controls [28]. Of note, this association was present >20 years before glioma diagnosis [28]. Epidemiological data are summarized in Table 1.

## 3. Allergic Mediators in Glioma and GBM

Much effort has been made to correlate the above described epidemiological observations with the prototypical biomarker of allergic inflammation, that is IgE. In 2004, Wiemels et al. found significantly lower IgE levels in glioma patients compared to controls (OR = 0.37, 95% CI, 0.22–0.64), with a more striking inverse association for IgE specific to food allergens (OR = 0.12, 95% CI, 0.04–0.41) [16]. However, a follow-up of this work by the same group has suggested that this inverse relationship is detectable only among cases receiving temozolomide [29]. Conversely, two later works have actually confirmed a relationship between serum IgE levels and gliomas. Indeed, a nested case-control study combining data from four prospective cohort studies have found a statistically significant inverse association between “borderline-elevated” total IgE levels (25–100 kU/L) and glioma (with 169 cases) (OR = 0.63, 95% CI, 0.42–0.93), even though no association was detected between high IgE (>100 kU/L) and glioma (OR = 0.98, 95% CI, 0.61–1.56) [30]. A prospective case-control study with a nested design including a higher number of cases (n = 275) has also demonstrated that the risk of glioma is inversely correlated to IgE response to inhalant allergens (OR = 0.73, 95% CI, 0.51–1.06) [31]. This relationship is particularly pronounced in women (OR = 0.53, 95% CI, 0.30–0.95) and the lowest OR was found in samples with the highest serum IgE levels [31]. Last, a nested case-control study with serum samples from 594 glioma and 374 GBM cases has shown that high levels of total IgE are associated with a significantly reduced risk of glioma, while allergen-specific IgE levels are correlated with a decreased risk of GBM specifically in women, but not in men [32]. Of note, this inverse association is present at least 20 years before tumor diagnosis [32].

A few studies have addressed the interplay between allergic inflammation and gliomagenesis with strategies different from the epidemiological approach. First experimental evidence can be found in initial attempts of immunotherapy. In a mouse model of glioma elicited in nude mice by the injection of U87 human glioma cells, the co-transplantation of an IL-4-secreting cell line promoted a significantly increased survival and a massive infiltration by eosinophils [33]. In line with these data, a phase I/II clinical trial has shown that the intracavitary injection of IL-2 and lymphokine-activated killer (LAK) cells in GBM patients leads to improved survival, associated with eosinophilic infiltration of the central nervous system (CNS) [34]. Several gene therapy approaches by our group and others have shown that IL-4 delivery in immunocompetent hosts with malignant gliomas results in a significant impairment of tumor growth and prolonged survival [35,36,37]. In particular, we found that local production of IL-4 at the tumor site is associated with increased infiltration of CD8^+^ T and CD4^+^ T cells, B lymphocytes and macrophages [37]. Later, we have shown that transplantation of neural stem/progenitor cells retrovirally engineered to produce IL-4 in C57BL/6 mice with established GBM leads to the survival of most tumor-bearing mice [38]. These data, obtained in both experimental models and human disease, suggest that the induction of an IL-4 and/or eosinophil-mediated immune response might be beneficial to counteract tumor development.

In addition, mast cells (MCs) have been implicated in the pathogenesis of glioma. MCs constitutively express the high affinity receptor for IgE (FcεRI) and represent the key effector players in allergic reactions, when the re-exposure to a previously encountered antigen induces IgE-mediated anaphylactic degranulation and the consequent release of massive amounts of pre-formed mediators, such as histamine, cytokines (e.g., tumor necrosis factor (TNF), IL-4), chemokines, leukotrienes and growth factors (e.g., vascular endothelial growth factor, VEGF) [39]. Furthermore, monomeric IgE in the absence of antigen, can be even more effective than IgE plus antigen to induce MC-mediated release of inflammatory cytokines such as IL-4, IL-6 and TNF [40]. Together with microglia, MCs represent a key immune subset resident within the CNS, where they are mainly localized in the thalamus, hypothalamus and leptomeninges [41]. Significantly increased numbers of MCs have been detected in GBM tissue specimens in comparison to low-grade glioma (grade II), displaying often a perivascular localization and staining for CXCL12 and CXCR4 [42]. Similar findings have been obtained in an immunocompetent mouse model of glioma, elicited with the retroviral system RCAS/TV-a of gene delivery. In detail, the RCAS retrovirus encoding for two oncogenes (*KRas* and *Akt*) has been administered postnatally in *Ntv-a* and *Gtv-a* transgenic mice, which express the TV-a receptor for RCAS retrovirus under the control of Nestin or GFAP promoters, respectively, to obtain retroviral infection (and consequent oncogene expression) specifically in neural/glial progenitor cells or astrocytes. These *Ntv-a* and *Gtv-a* mouse lines carried at the same time deficiencies in tumor suppressor genes *Ink4a* or *Arf*, which are often deleted in human gliomas and developed gliomas of various grades and types [42]. The mouse strain with high-grade glioma (*Arf*^−/−^) displayed a significantly higher infiltration of MCs than *Ink4a*^−/−^ mice developing low-grade gliomas and exhibited also a strong expression of stem cell factor (SCF), the main growth factor for MCs, around tumor blood vessels [42]. Interestingly, primary human gliomas have been shown to express SCF in a grade-dependent manner [43]. The same research group has later extended these findings to a larger cohort of patients (n = 188), confirming an accumulation of MCs in high-grade (i.e., grade III and IV) vs. low-grade (grade II) gliomas [44]. By taking advantage of a transwell migration assay, human glioma cell lines were shown to potently attract MCs by a mechanism partially dependent on the chemoattractant macrophage migration inhibitory factor (MIF), plasminogen activator inhibitor 1 (PAI-1) and the phosphorylation of STAT5 [44,45]. Of note, a significantly positive correlation was observed between the number of MCs and the cytoplasmic intensity of MIF staining in glioma tissue samples [44].

Notably, a recent analysis from lower grade gliomas showed that allergy is a robust, prognostic factor independent from other major prognostic molecular markers [46].

## 4. Conclusions and Future Perspectives

**Figure 1:**
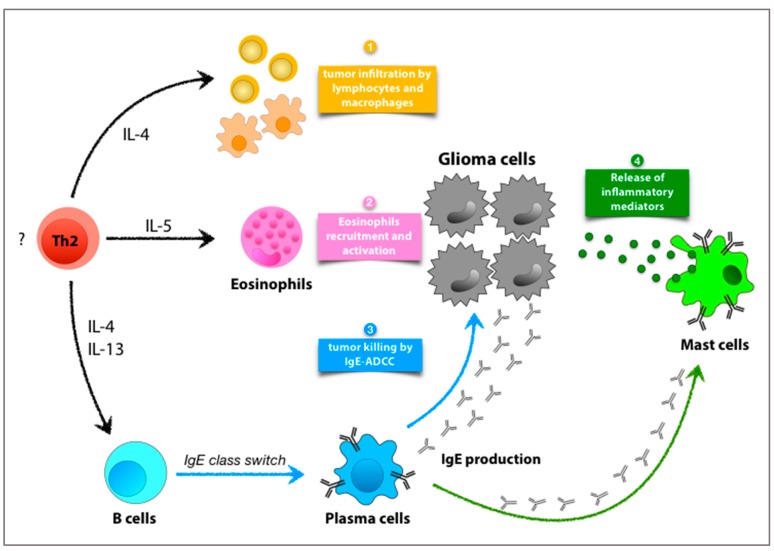
Schematic representation of the components of allergic inflammation with potentially anti-glioma activity. The establishment of a Th2-driven immune response against the tumor might support resistance to glioma growth through several modalities. The overexpression of the prototypical Th2-cytokine interleukin (IL)-4 within tumor microenvironment has been shown to counteract gliomagenesis [33,35,36,37,38,47]. This anti-tumor effect is associated with enhanced infiltration of immune cells such as CD8^+^ T lymphocytes, CD4^+^ T cells, B cells and monocytes at the tumor site (1) [37]. Th2 cells also secrete IL-5, a key cytokine for the recruitment and activation of eosinophils. Eosinophilic infiltrates within glioma tissue have been detected in early attempts of immunotherapy displaying a positive outcome [33,34] (2). IL-4 and IL-13 derived from Th2 cells can promote IgE isotype class switch recombination in B cells and their maturation to plasma cells. Theoretically, antigen-specific IgE might promote direct tumor killing through ADCC (3), similar to what was observed in a mouse model of ovarian cancer [48]. Alternatively, the engagement of FcεRI on mast cells (MCs) by monomeric IgE is known to induce the release of selective inflammatory cytokines [40], such as IL-4 and tumor necrosis factor (TNF), both endowed with anti-glioma activity [38,49] (4).

Type 2 immunity is recently emerging as a specific arm of the immune system physiologically deputed to defense against toxic and carcinogenic agents [5]. Data so far have suggested that at least some components of allergic inflammation might counteract the development of gliomas and GBM (Figure 1).

Epidemiological investigations have provided a huge amount of data showing an inverse association between allergy and gliomas, even though they do not provide any information on the causative relationship between these two conditions. In principle, it is possible that the tumor itself promotes immune-suppression of type 1 immunity and indirectly favors an imbalance of systemic immunity toward a Th2/allergic profile. However, the timing of most epidemiological observations, anticipating the clinical emergence of gliomas of many years, is in some contradiction with this hypothesis, at least in cases of primary GBM that are known to be formed in months rather than years. Moreover, some genetic variants in *IL13* and *IL4RA* genes confer opposite odds ratios for asthma and GBM development [27]. Given that germline polymorphisms were utilized as biomarkers of susceptibility to asthma in this study, these results cannot be ascribed to an effect of GBM on the immune system [27]. According to the cancer immunoediting hypothesis, the interaction between the immune system and tumor cells undergoes three different stages termed respectively “elimination”, “equilibrium” and “escape” [50]. The elimination phase is characterized by the interplay of innate and adaptive immune mechanisms, which drives the destruction of tumor cells from the host. However, if this process is not complete, rare cancer cell variants might enter an equilibrium stage, in which tumor growth is still prevented by adaptive immunity and the tumor is subject to constant editing of its immunogenicity. During this stage, immune pressure on genetically-unstable tumor cells might result in the selection of tumor variants endowed with the ability to escape immune surveillance and form clinically visible tumors [50]. In the context of brain tumors, it’s possible to speculate that the genetic background favoring the development of allergic inflammation might support the elimination phase and reduce the probability of developing glioma over the course of time.

Immunotherapeutic approaches have shown that IL-4 can counteract glioma growth and eosinophilic infiltration of tumor tissue correlates with a positive outcome of immunotherapy. Nonetheless, the mechanisms through which these typical components of allergic inflammation might exert an anti-tumoral effect are not clearly understood.

Higher levels of IgE are associated with a reduced risk of glioma. IgE is endowed with tumor-killing properties by mediating antibody-dependent cell-mediated cytotoxicity. Its effect has been shown in a mouse model of ovarian cancer [48], but no study has investigated whether IgE is reactive against tumor antigens in glioma patients. Furthermore, IgE alone or in combination with an antigen can stimulate MC functions.

Studies describing the accumulation of MCs in GBM have offered important evidence of the potential involvement of these crucial effector cells of allergy in gliomagenesis, however, whether MCs promote or counteract tumor growth is not known and should be subject to further investigation. According to the modality of activation, MCs have been shown to promote or break immune tolerance. This concept is illustrated in models of allotransplantation and CD8^+^ T cell-mediated graft rejection. Indeed, IL-9 secreted by regulatory T (Treg) cells has been shown to recruit and activate MCs to promote regional immune suppression and acceptance of allograft skin transplants [51]. Conversely, in the same model when MCs undergo anaphylactic degranulation, they break Treg-mediated suppression and induce allograft rejection [52]. To understand the contribution of MCs to gliomagenesis, it might be worthwhile to evaluate the development of gliomas in mouse models of MC-deficiency [53]. Interestingly, recent work has shown that *Hdc*^−/−^ mice, that genetically lack histamine, exhibit increased glioma growth and reduced survival, associated with the accumulation of immunosuppressive immature myeloid cells [54]. Of note, MC granules represent the main immunological source of histamine and *Hdc*^−/−^ mice display several deficits in MC numbers, morphology and granular contents [55]. In addition, there are data supporting the contribution of MCs to tumor formation in the context of neurofibromatosis type 1 [56].

## Figures and Tables

**Table 1:** Epidemiological studies of allergy and risk of glioma.

Study Type	Number of Glioma Cases	Number of Control Subjects	Association with Allergy (95% CI)	Year [Ref.]
Case-control	115	418	RR = 0.71 (0.5–1.0)	1992 [13]
Case-control	1178	2493	RR = 0.59 (0.49–0.71)	1999 [14]
Case-control	405	402	OR = 0.5 (0.3–0.7)	2002 [15]
Case-control	489	799	OR = 0.67 (0.52–0.86)	2002 [17]
Cohort study	Cohort I (14535 subjects/33 glioma cases)		HR = 0.38 (0.05–3.13) ^1^ HR = 0.46 (0.18–1.21) ^2^	2003 [18]
	Cohort II (29573 subjects/42 glioma cases)		HR = 2.60 (0.86–7.81) ^1^ HR = 0.45 (0.11–1.92) ^2^	
Case-control	965	1716	OR = 0.63 (0.53–0.76)	2006 [19]
Meta-Analysis	Participants (53223 subjects/3450 glioma cases)		RR = 0.61 (0.55–0.67)	2007 [20]
Case-control	1527	3309	OR = 0.70 (0.61–0.80)	2007 [21]
Case-control	366	1494	OR = 0.92 (0.70–1.22)	2009 [22]
Case-control	388	80 (siblings)	OR = 0.53 (0.15–1.84)	2009 [23]
		191 (friends)	OR = 0.54 (0.28–1.07)	
		177 (clinic-based controls)	OR = 0.34 (0.23–0.50)	
Case-control	855	1160	OR = 0.62 (0.51–0.76)	2011 [24]
Cohort study	4.5 million subjects/4383 malignant neoplasm brain ^3^		Rate ratio = 0.60 (0.43–0.83)	2014 [25]
Case-control	273	982	OR = 0.52 (0.36–0.75)	2018 [26]

Confidence interval (CI); relative risk (RR); odds ratio (OR); hazard ratio (HR). ^1^ Association of low-grade (I and II) glioma cases. ^2^ Association of high-grade (III and IV) glioma cases. ^3^ Brain tumors included rare childhood tumors, but mainly gliomas, expected to cover 95% of cases. Ref., reference.
